# Sustainable superhydrophobic coating based on in-situ electrodeposited Ni-Al layered double hydroxide for enhanced corrosion protection of steel

**DOI:** 10.1038/s41598-026-44678-z

**Published:** 2026-04-11

**Authors:** D. M. Ragheb, M. M. Zaki, F. M. Mahgoub, W. A. Sadik, M. E. Mohamed

**Affiliations:** 1 Materials Science Department, Institute of Graduate Studies & Research, Alexandria University, Alexandria, Egypt; 2 Chemistry Department, Faculty of Science, Alexandria University, Alexandria, Egypt; 3 Faculty of Advanced Basic Sciences, Alamein International University, Alamein City, Matrouh Governorate Egypt

**Keywords:** Superhydrophobic; Layered Double Hydroxide, Corrosion Protection, Steel, Electrodeposition, Hierarchical Structure, Chemistry, Engineering, Materials science, Nanoscience and technology

## Abstract

The development of durable superhydrophobic coatings for corrosion protection remains a significant challenge due to the weak mechanical and chemical stability of most synthetic surfaces. This work presents a highly stable, eco-friendly superhydrophobic coating fabricated on steel via a facile two-step process. Ni-Al layered double hydroxide (LDH) with a unique Micro-nano morphology was first grown in-situ by a one-pot electrodeposition method, creating a rough hierarchical structure. This was subsequently functionalized with stearic acid, a green low-surface-energy material, to achieve superhydrophobicity. Systematic optimization of the electrodeposition parameters revealed that a 15-minute deposition at 40 mA yielded the optimum coating, which exhibited a water contact angle of 161° and a sliding angle of 3°. Extensive characterization, including SEM, AFM, XRD, and XPS, confirmed the successful formation and structure of the coating. Electrochemical tests in a solution of 0.5 M NaCl displayed that the optimized coating significantly enhanced the corrosion resistance of steel, achieving a protection efficiency of 96.5%. Crucially, the coating exhibited exceptional durability, maintaining its superhydrophobicity after 1100 mm of linear abrasion and demonstrating remarkable chemical stability against corrosive media across a wide pH range from 1 to 13. This study not only provides a novel method for constructing environmentally stable LDH-based coatings but also highlights their significant potential for real-world anti-corrosion applications in demanding environments.

## Introduction

 The degradation of steel through corrosion represents a significant global economic and safety challenge, particularly in industries such as marine infrastructure, chemical processing, and energy, where exposure to aggressive chloride-containing environments is prevalent^1,2^. While traditional anti-corrosion strategies like corrosion inhibitors and polymeric coatings are effective, they often face limitations regarding environmental impact, long-term durability, and complex application procedures^3,4^. Consequently, the development of novel and efficient protective coatings remains a critical research objective.

In recent years, bio-inspired superhydrophobic surfaces have attracted substantial interest as an efficient approach for corrosion protection. These surfaces are characterized by a water contact angle (WCA) above 150° and a water sliding angle (SA) below 10°, they function by trapping a stable air film within a hierarchical micro/nanoscale surface texture, which acts as a physical barrier to isolate the underlying metal from the corrosive electrolyte^5^. The fabrication of such surfaces universally requires two fundamental criteria: constructing a suitable surface roughness and subsequent chemical modification with a low-surface-energy material^6^.

A wide array of techniques has been explored to create the necessary surface roughness for superhydrophobicity. These include chemical etching^7^, sol-gel processing^8^, chemical vapor deposition^9^, spray coating^10^, and electrochemical methods^11^. Among these, electrochemical deposition is particularly favorable as it offers facile control and high reproducibility in creating surface roughness, along with mild operating conditions and scalability^12^.

Layered Double Hydroxides (LDHs) have garnered considerable attention as versatile nano-structured materials for constructing this necessary roughness^13,14^. LDHs represent a class of ionic lamellar compounds with the typical formula [M^2 +^_1−x_ M^3 +^_x_(OH)_2_]^x+^(A^n−^)_x/n_·mH_2_O. In this structure, divalent (e.g., Zn^2+^) and trivalent (e.g., Al^3+^) metal cations form positively charged brucite-like layers. The interlayer region of LDH structures comprises charge-balancing anions (A^n−^), such as NO_3_^−^ or CO_3_^2−^, together with intercalated water molecules (m), which occupy the interlayer galleries and contribute to structural stability^13^. Their unique two-dimensional layered structure, tunable chemical composition, and anion exchange capacity make them ideal candidates for corrosion-resistant coatings. LDH offers the distinct advantage due to its ability to form dense, adherent, and conformal coatings with precisely controlled nano-architectures directly on conductive substrates. The resulting roughness is integral to the substrate, promoting excellent adhesion and durability compared to methods that simply deposit pre-formed particles^14,15^. The electrodeposition of LDHs has recently gained significant attention for developing advanced functional materials. While this technique has been leveraged for energy storage (e.g., NiMn-LDH on MOFs for supercapacitors^16^ and catalysis (e.g., NiFe-LDH for seawater splitting^17,18^, its potential for corrosion protection is particularly promising. Pioneering work by Dong et al. demonstrated that electrodeposited LDH films intercalated with inhibitor anions could achieve corrosion inhibition efficiencies greater than 97%^19^, validating the approach of using LDHs as a barrier layer. The present work builds upon this foundation by exploring a Ni-Al LDH system for creating a superhydrophobic surface, adding a passive physical barrier (air) to the active corrosion inhibition offered by the LDH structure.

Following roughness construction, the surface energy must be lowered to achieve water repellency. Historically, long-chain perfluorinated compounds (PFCs) like fluorosilanes have been the material of choice due to their exceptionally low surface energy^6,20^. However, growing environmental and health concerns regarding the persistence and toxicity of synthetic fluorocarbon compounds have driven the search for eco-friendly alternatives. In this context, naturally sourced, long-chain fatty acids, for instance stearic acid, have gained prominence as sustainable and effective low-surface-energy materials. Stearic acid, with its carboxylic acid head group that readily chemisorbs onto metal oxides/hydroxides and its long hydrocarbon tail, provides a stable hydrophobic coating, making it an ideal green candidate for fabricating superhydrophobic surfaces^21^.

The potential applications of superhydrophobic coatings extend far beyond corrosion protection. They are actively researched for anti-icing on aircraft and power lines^22,23^, self-cleaning windows and solar panels, oil-water separation^24^, and drag reduction in marine and microfluidic applications^25,26^. However, for many of these real-world applications, two persistent challenges must be overcome: insufficient mechanical stability and poor chemical stability in extreme environments. The insufficient mechanical stability of the SHP coatings is arises because their delicate micro-nano structures are easily damaged by abrasion or scratching, leading to loss of water repellency and accelerated localized corrosion at defect sites^20,26^. While numerous methods can create superhydrophobic surfaces, many result in fragile morphologies susceptible to abrasion or degradation in harsh chemical environments, severely limiting their practical utility. This work bridges this gap by utilizing an in-situ electrodeposition process to fabricate a Ni–Al LDH coating directly on the steel substrate. This method produces an integrated, rigid micro-nano structure that is inherently more stable than deposited particulates or etched surfaces. The strong adhesion and cohesive strength of this electrodeposited LDH provides the mechanical foundation, while its chemical stability forms a resilient scaffold for subsequent modification. To the best of our knowledge, this represents one of the first reports on the in-situ electrodeposition of an LDH specifically for constructing a superhydrophobic surface with the explicit aim of achieving long-term corrosion protection, offering a novel and viable pathway to overcome the durability bottleneck in the field.

This work aims to construct an environmentally stable superhydrophobic coating for steel through the strategic combination of an electrodeposited Ni-Al LDH nano-layer and a stearic acid modification. The novelty of this research lies in its integrated approach: it utilizes a one-pot electrodeposition process to create a strong adherent, micro-nano LDH roughness and exploits the green alternative of stearic acid for low-surface-energy functionalization. Furthermore, this study provides a comprehensive evaluation of the coating’s performance, with a specific focus on its corrosion resistance, mechanical and chemical stability—key metrics for real-world applications that are often overlooked. The LDH coat was characterized using Energy Dispersive X-ray Spectroscopy (EDX) to elucidate its morphology and elemental composition, while X-ray Diffraction (XRD) and X-ray Photoelectron Spectroscopy (XPS) were employed to determine its crystal structure and chemical state. The final superhydrophobic coated steel was analyzed using Scanning Electron Microscopy (SEM) and Atomic Force Microscopy (AFM) to verify the retention of hierarchical surface roughness following functionalization, and its wetting behavior was quantitatively assessed through WCA and SA measurements. This work thus establishes a new pathway for designing durable, environmentally benign superhydrophobic coatings with enhanced practical viability.

## Material and method

### Materials

Carbon steel sheets (20 × 50 × 2 mm^3^) and aluminum sheets (20 × 50 × 2 mm^3^) were employed as substrates in this study. Ni (NO_3_)_2_.6H_2_O, NaOH, stearic acid, anhydrous ethanol (99.9%), sulfuric acid, and NaCl were purchased from Central drug house (CDH) Co., Ltd. During the preparation of chemical solutions, a magnetic stirrer was operated at 500 rpm, and all solutions were synthesized via double-distilled water.

### Construction of superhydrophobic Ni-Al LDH coat on steel substrates

Prior to electrodeposition, the steel substrate was insulated with an epoxy resin, leaving a precise working area of 2 cm^2^ uncovered to the electrodeposition bath. The steel specimens were abraded mechanically with silicon carbide (SiC) of varying grades (240, 500, and 1000) for removing oxide. Subsequently, the polished substrates were ultrasonically cleaned in absolute ethyl alcohol.

Ni-Al LDH coatings were fabricated on steel substrates via an electrochemically-induced co-precipitation method using a two-electrode configuration. The process relies on cathodic nitrate reduction to generate a localized alkaline environment, which triggers co-precipitation of Ni^2+^ions from the electrolyte and Al^3+^ ions generated by anodic dissolution of the aluminum counter electrode. This results in direct formation of the Ni-Al LDH film on the steel cathode without reduction of Al^3+^ to metallic aluminum. This approach is consistent with previously reported electrodeposition methods for fabricating Al-containing LDH films^17,27–29^. A carbon steel coupon served as the cathode, while an aluminum sheet of same dimensions was used as the sacrificial anode. The two electrodes were positioned parallel to each other with a fixed inter-electrode distance of 2.0 cm.

The electrolyte solution was prepared by dissolving 0.5 M nickel nitrate hexahydrate (Ni(NO_3_)_2_·6H_2_O) in double distilled water. The solution pH was adjusted to 8.0 using a 2.0 M sodium hydroxide solution. Electrodeposition was performed under galvanostatic (constant current) conditions. The deposition process was optimized by varying the deposition time (7.5, 15, and 22.5 min) at a fixed optimum current density of 40 mA, which was determined from preliminary experiments.

Following electrodeposition, the coated steel substrates were rinsed with distilled water to eliminate residual salts and dried in ambient air for 24 h. To impart superhydrophobicity, the dried LDH coated samples were submerged in an ethanolic solution of 0.01 M stearic acid for 15 min. The samples were subsequently immersed in ethanol to eliminate any physisorbed stearic acid molecules and then dried at room conditions for 24 h. Both the pristine LDH coating and the stearic acid-modified LDH coating (LDH–SA) were subjected to comprehensive evaluation and characterization.

The advantages of the one-pot electrodeposition technique:

- **In-Situ Al**^**3+**^ **generation**: The sacrificial aluminum anode provides continuous, controlled supply of trivalent cations, eliminating the need for aluminum salts in the electrolyte and simplifying bath composition.
- **Precise morphological control**: Galvanostatic conditions enable fine-tuning of coating thickness and surface architecture through simple adjustment of deposition time and current density.
- **Superior substrate adhesion**: Direct growth of LDH on the steel substrate ensures environmentally stable interfacial bonding without requiring binders or post-synthesis transfer.
- **Energy and time efficiency**: One-pot processing reduces energy consumption and fabrication time compared to multi-step hydrothermal or co-precipitation methods.
- **Green functionalization**: Subsequent modification with stearic acid represents an environmentally benign alternative to hazardous fluorinated compounds commonly used for superhydrophobicity.
- **Scalability**: The electrochemical approach is readily adaptable to large-scale or continuous processing of metallic substrates with complex geometries.
- **Cost-effectiveness**: Minimal chemical consumption and ambient processing conditions reduce overall fabrication costs.

### Characterization

#### Characterization of the LDH coat

The elemental analysis of the LDH coat was performed by EDX via GEM-2100 instrument from JEOL. The crystal structure of the LDH layers was analyzed using X-ray diffraction (XRD, Germany, λ = 0.15406 nm, Bruker, D8 Advance) at a scanning rate of 4° min^− 1^ within the range of 2θ = 5–90°. The Ni-Al LDH phase was identified based on its characteristic hydrotalcite-like structure, with peak positions consistent with JCPDS card 15–0087 for Ni-Al LDH, as well as with previously reported electrodeposited LDH phases in the literature^30–33^.

The chemical composition and elemental states were investigated via X-ray photoelectron spectroscopy (XPS), via Thermo Fisher Scientific. The EDX, XRD, and XPS analysis were performed for the optimum deposited Ni-Al LDH coat.

#### Characterization of the superhydrophobic LDH coat

The superhydrophobicity of the modified LDH film was assessed by measuring the WCA and SA via Ramé-Hart Goniometer instrument (Model 190) with DROP image CA v2.5. The morphology of the surface and microstructure were examined by SEM via JSM- IT200 instrument. The surface topography and roughness were inspected by AFM using Scanning Probe Microscope (SPM9600—Shimadzu Japan).

The corrosion behaviour of the LDH-SA coating at different deposition times (7.5, 15, and 22.5 min) was evaluated using electrochemical impedance spectroscopy (EIS) and potentiodynamic polarization measurements accomplished with a potentiostat (PARSTAT, USA). All electrochemical measurements were performed via a conventional three-electrode system, involving a platinum counter electrode and an Ag/AgCl reference electrode. The tests were performed in a solution of 3.5 wt% NaCl under room conditions. Prior to testing, both bare steel and LDH-SA-coated steel samples were submerged in the electrolyte for 20 min to stabilize the open circuit potential (OCP).

Impedance measurements were recorded from 100 kHz to 0.01 Hz with an applied potential signal amplitude of 10 mV around the rest potential. Potentiodynamic polarization curves were recorded from − 350 mV to + 350 mV relative to the open circuit potential (OCP) at a scan rate of 30 mV min^− 1^. This extended range was employed to investigate the corrosion mechanism, including diffusion-controlled behavior in the cathodic region and possible passivation in the anodic region. Tafel extrapolation for corrosion parameters was performed on the linear regions within ± 250 mV of OCP in accordance with ASTM G59 guidelines. To ensure accuracy, all experiments were repeated, and the results were confirmed to be within a 2% error margin. All electrochemical measurements were performed in triplicate to ensure reproducibility. The reported parameters represent mean values ± standard deviation calculated from three independent measurements.

The mechanical durability of the optimized LDH-SA coating (deposited for 15 min) was assessed using scratch tests. For this purpose, the coated samples were abraded against 1000-grit SiC paper under a pressure of 2.5 kPa. The water contact angle (WCA) and sliding angle (SA) of water droplets on the LDH-SA coating were estimated after every 50 mm of abrasion. Additionally, the chemical stability of the optimized LDH-SA coating was assessed by dipping the samples for 30 min in solutions with varying pH values (pH 1–13). The solutions pH was adjusted using 0.5 M sulfuric acid and 0.5 M sodium hydroxide and WCA and SA were recorded after each immersion. The error bars in Figs. 10 and 12 represent standard deviation from replicate experiments.

## Results and discussion

### Formation and the characterization of the Ni-Al LDH

#### Formation of the Ni-Al LDH

The primary finding is the successful fabrication of a Ni-Al LDH coating on a steel substrate via a one-pot electrodeposition method. The process relies on the electrochemical generation of the trivalent metal ion (Al^3+^) in situ. At the anode (Al sheet), oxidation occurs:

1 $${\text{Al }} \to {\text{ A}}{{\mathrm{l}}^{{\mathrm{3}}+}}+{\text{ 3}}{{\mathrm{e}}^ - }$$

This is the crucial outcome. At the cathode, the reduction reaction of nitrate ions (NO_3_-) consumes electrons and water and produces hydroxyl ions (OH^-^). This leads to a pronounced local increase in pH at the steel cathode surface:

2 $${\mathrm{N}}{{\mathrm{O}}_{\mathrm{3}}}^{ - }+{{\mathrm{H}}_{\mathrm{2}}}{\mathrm{O}}\,+\,{\mathrm{2}}{{\mathrm{e}}^ - } \to {\text{ N}}{{\mathrm{O}}_{\mathrm{2}}}^{ - }+{\text{ 2O}}{{\mathrm{H}}^ - }$$

The metal ions (Ni^2+^ from the solution and Al^3+^ generated at the anode) are attracted to the negatively charged cathode. When they encounter this localized, high-pH environment, they can no longer remain soluble and co-precipitate as a solid Ni-Al LDH on the steel surface.

The overall process can be summarized as:

3 $${\mathrm{xN}}{{\mathrm{i}}^{{\mathrm{2}}+}}+{\text{ yA}}{{\mathrm{l}}^{{\mathrm{3}}+}}+{\text{ }}\left( {{\mathrm{2x}}\,+\,{\mathrm{3y}}} \right){\mathrm{O}}{{\mathrm{H}}^ - }{\text{ }} \to {\text{ N}}{{\mathrm{i}}_{\mathrm{x}}}{\mathrm{A}}{{\mathrm{l}}_{\mathrm{y}}}{\left( {{\mathrm{OH}}} \right)_{{\mathrm{2}}x+{\mathrm{3}}\gamma }}\left( {{\text{as a solid film}}} \right)$$

#### Characterization of the Ni-Al LDH

**Fig. 1:**
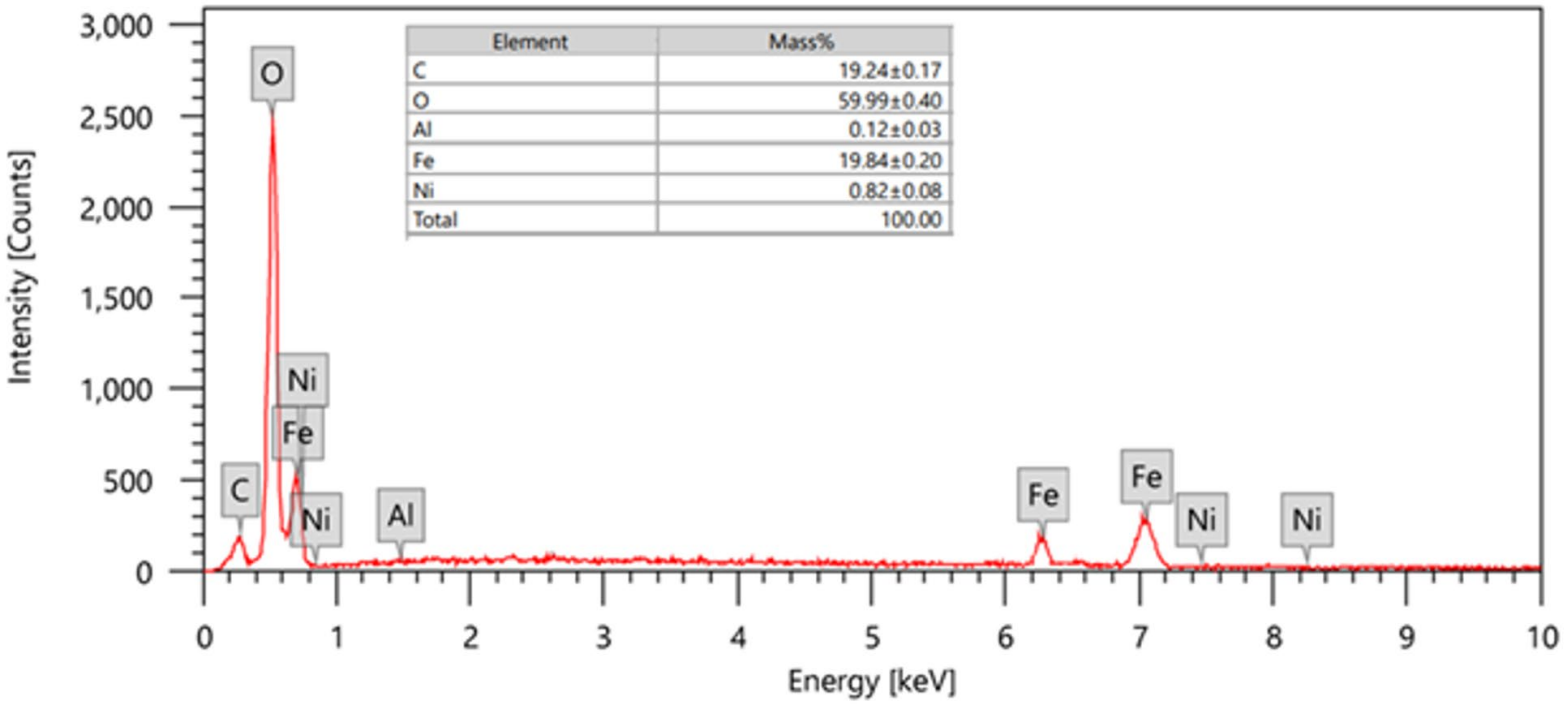
EDX spectra of the electrodeposited Ni-Al LDH.

The EDX spectrum, Fig. 1, shows distinct peaks for Ni, Al, O, and the absence of other major metallic contaminants provides strong evidence for the formation of the intended Ni-Al LDH. While the detected presence of carbon is likely attributable to atmospheric CO_2_, which LDHs are known to intercalate as carbonate anions, further confirming the LDH structure. The Fe signal confirms that the analysis probed through to the substrate, verifying that the coating is a thin layer.

**Fig. 2:**
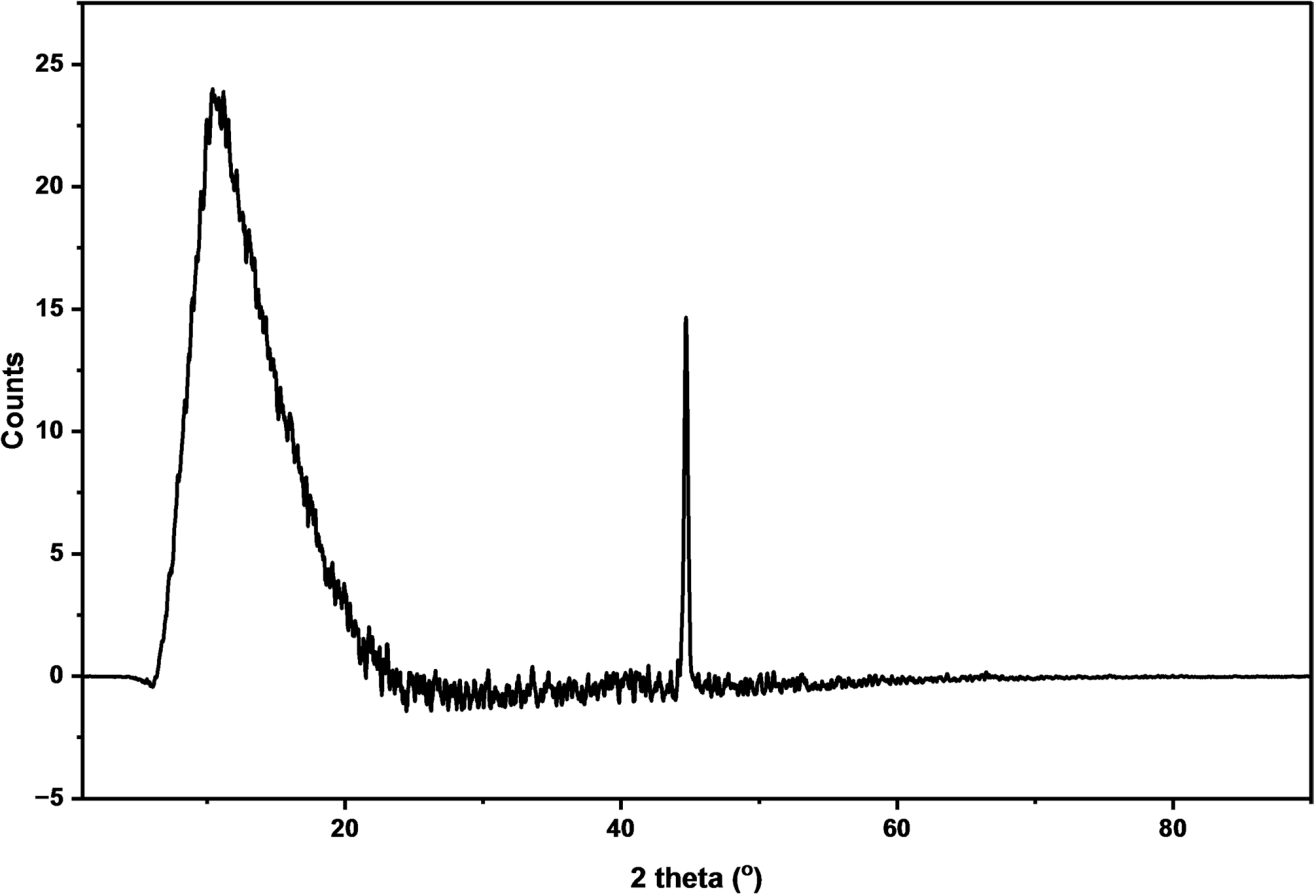
XRD spectra of the electrodeposited Ni-Al LDH (JCPDS 15–0087)^30–33^.

Figure 2 reports the X-ray diffraction (XRD) pattern of the synthesized Ni–Al LDH. The pattern is characterized by a broad reflection at 2θ of 16° and a sharper peak at 2θ of 45°, which are indicative of a nanocrystalline and turbostratic LDH structure. The broad peak at 16° is ascribed to the (003) basal plane reflection. Its position suggests the intercalation of carbonate anions within the LDH interlayers, a common phenomenon due to the affinity of LDHs for atmospheric CO_2_^34,35^. The significant broadening of this peak is attributed to the small crystalline size and disorder in the stacking direction of the LDH layers, which is a typical outcome of the rapid electrodeposition process. The sharper peak at 45° corresponds to the (018) reflection, related to the in-plane ordering within the brucite-like layers. The XRD analysis confirms the successful formation of a Ni-Al LDH phase. The observed nanocrystalline and potentially turbostratic nature of the electrodeposited LDH is, in fact, advantageous for the application as a superhydrophobic coating.

**Fig. 3:**
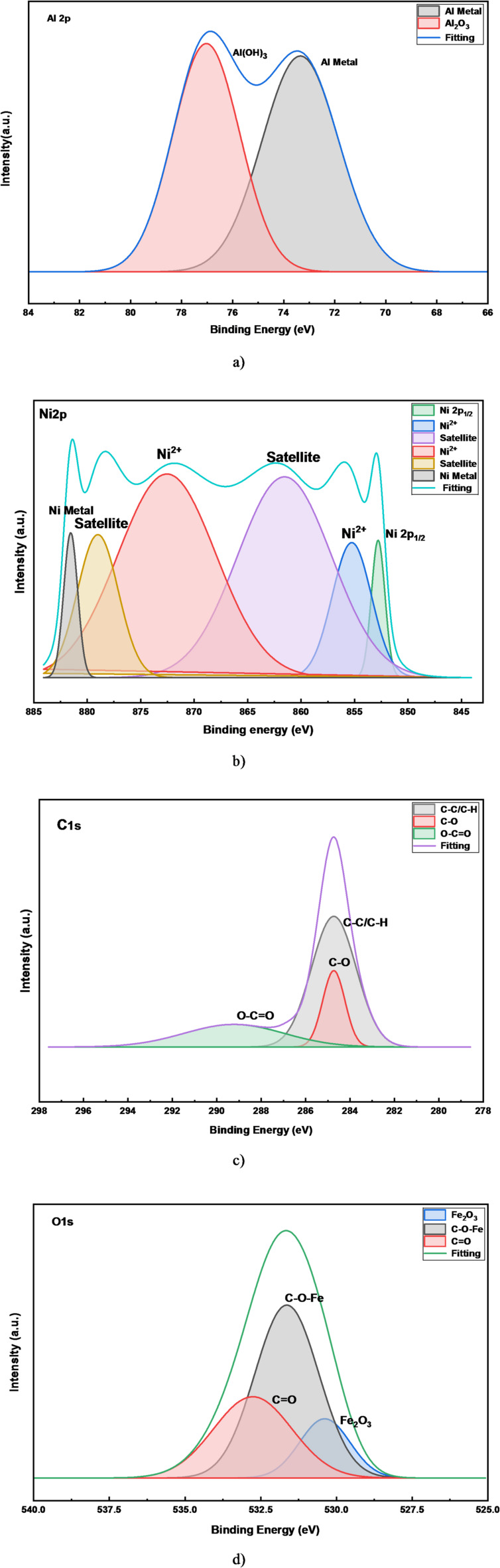

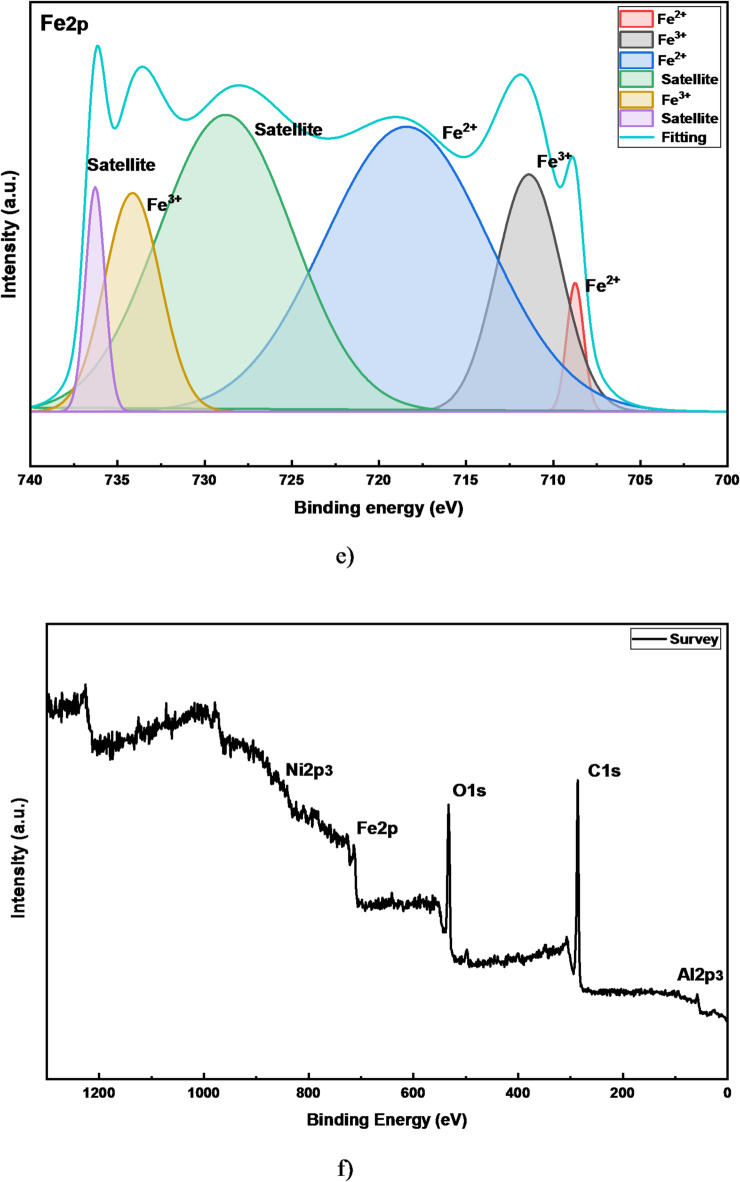
High-resolution XPS spectra of the electrodeposited Ni-Al LDH, **a**) Aluminum spectrum, **b**) Nickel spectrum, **c**) Carbon spectrum, **d**) Oxygen spectrum, **e**) iron spectrum and **f**) Survey of all elements.

The XPS data confirms the effective synthesis of the Ni-Al LDH and provides deep insight into the chemical bonding and composition of the prepared LDH. For Aluminum spectrum, Fig. 3a, the presence of Al (OH)_3_ is the primary finding. In LDH, aluminum exists in the + 3 oxidation state coordinated by hydroxyl groups. The Al metal peak could be due to a very small amount of non-oxidized Al from the anode that was incorporated. The high-resolution Ni 2p XPS spectrum, Fig. 3b, provides definitive evidence for the chemical state of nickel within the coating. The presence of intense shake-up satellite peaks at approximately 861.5 eV and 879.5 eV is a characteristic fingerprint of paramagnetic, high-spin Ni^2+^. These satellites, coupled with the corresponding main peaks at 855.7 eV (Ni 2p_3_/_2_) and 873.0 eV (Ni 2p₁/₂), unequivocally confirm that nickel exists predominantly in the + 2 oxidation state within a Ni(OH)_2_-like coordination environment. This finding is consistent with the successful formation of the nickel-aluminum LDH phase, where Ni^2+^ constitutes the primary cation in the brucite-like layers. The minor peak observed at 881.2 and 852.5 eV is attributed to a trace amount of metallic nickel (Ni⁰), likely resulting from the electrodeposition process, but it does not constitute a dominant phase. For Carbon (C 1s) spectrum, Fig. 3c, three peaks identified: C-C/C-H, C-O, O-C = O. This spectrum reveals the sources of carbon contamination and the nature of intercalated anions. C-C/C-H: This is the main component of adventitious carbon; ubiquitous hydrocarbon contamination found on all surfaces exposed to air. This is standard and used for charge referencing. C-O & O-C = O: These are the critical peaks. They provide strong evidence for the intercalation of carbonate (CO_3_^2−^) anion in the LDH gallery. The O-C = O peak is characteristic of carbonate. This perfectly explains the XRD peak shift at 2θ ~ 16°. The C-O could also be from carbonate. For Oxygen (O 1s) spectrum, Fig. 3d, shows three peaks: Fe_2_O_3_, C-O-Fe, C = O. This is a complex spectrum that confirms the composite nature of the interface. Fe_2_O_3_: This signal comes from the steel substrate as the deposited LDH layer is very thin. This correlates with the Fe signal in EDX. C-O-Fe: This suggests a chemical interaction between the steel substrate and the carbonaceous species (potentially from the electrolyte), indicating some level of bonding at the interface. The C = O peak is attributed to carbonate (CO_3_^2−^) anion in the LDH. For iron (Fe 2p) spectrum, Fig. 3e, the present peaks are: Fe^2+^, Fe^3+^, and their satellites. This unambiguously confirms that the iron signal originates from the oxidized steel substrate (likely a mix of Fe_2_O_3_ and Fe_3_O_4_), not from metallic iron. The presence of satellite peaks is characteristic of iron oxides. Figure 3e shows the survey of all elements present in the sample. Figure 3F shows the survey of all elements.

The XPS data confirms that:

1. LDH formation confirmed: The chemical states of Ni (Ni^2+^ in an hydroxide environment) and Al (Al^3+^ in a hydroxide environment) are definitive fingerprints of the LDH structure.
2. Intercalated anions identified: The presence of carbonate anions (from C 1s and O 1s spectra) explains the interlayer spacing observed in XRD and is consistent with the affinity of LDHs for atmospheric CO_2_.
3. Overall Consistency: The XPS data is in full agreement with the EDX and XRD results, painting a consistent picture of a nanocrystalline, carbonate-intercalated Ni-Al LDH coating on steel.

### Characterization of the prepared LDH-SA coats

**Fig. 4:**
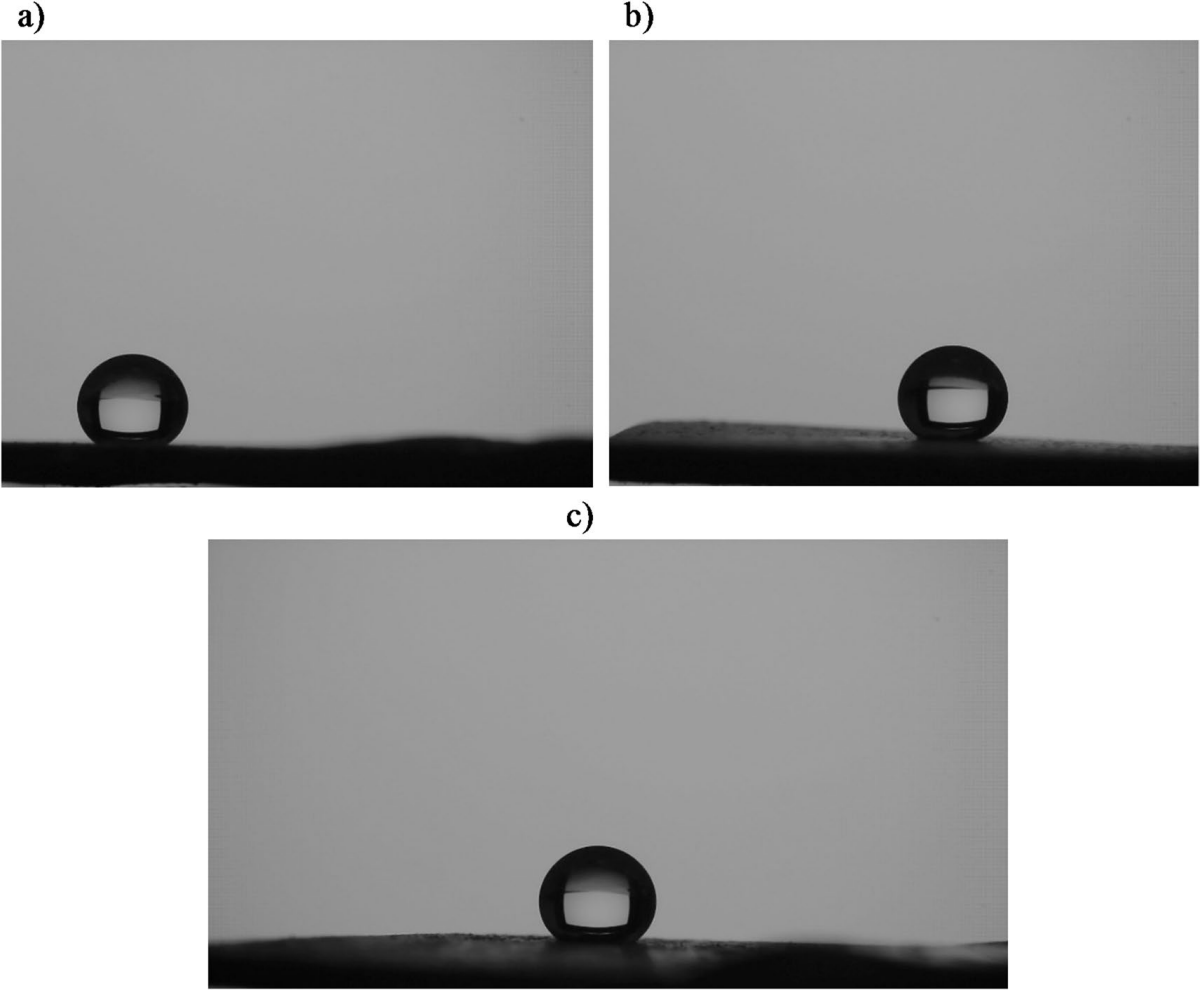
Images of water droplets on the LDH-SA coatings electrodeposited for (a) 7.5, (b) 15, and (c) 22.5 min.

The superhydrophobic performance was optimized at a deposition time of 15 min, which yielded a WCA of 161° and a SA of 3°. This represents the highest repellency and lowest adhesion, as the shorter (7.5 min, WCA = 159°, SA = 4°) and longer (22.5 min, WCA = 156°, SA = 6°) deposition times resulted in degraded performance. The surface morphology and roughness, examined using SEM and AFM, provide structural insights into the observed wettability behavior. Figure 4 illustrates the water droplet profiles on LDH-SA coatings prepared at different deposition times.

**Fig. 5:**
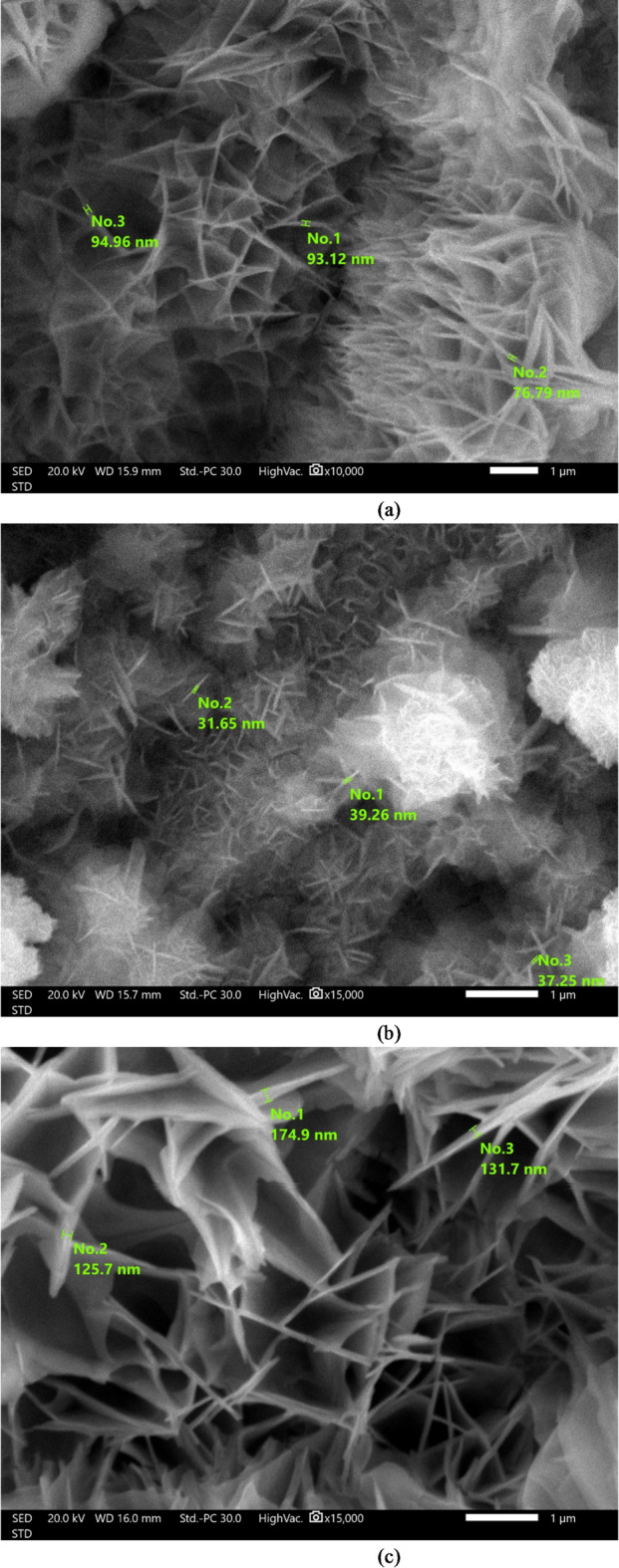
SEM Micrographs of the LDH-SA coatings produced at different deposition times, for (**a**) 7.5, (**b**) 15, and (**c**) 22.5 min.

The surface morphology was inspected using SEM technique, Fig. 5. The surface morphology of the LDH-SA coatings evolves significantly with electrodeposition time, shifting from nanowalls to nanoneedles and back to larger nanowalls, which directly dictates their superhydrophobic performance. At the sub-optimal 7.5-minutes deposition time, an under-developed structure of fine nanowalls (average size ~ 88 nm) forms, providing moderate roughness but incomplete surface coverage. The optimum 15-minutes deposition, however, results in a distinct and highly effective nanoneedle structure with a fine thickness of ~ 36 nm. This sharp, high-aspect-ratio morphology is superior for creating a hierarchical texture, maximizing the number of nanoscale air-trapping pockets. In contrast, the 22.5-minutes deposition leads to significant overgrowth via Ostwald ripening, transforming the structure back into coarse nanowalls with a much larger average size of ~ 144 nm. This overgrowth smoothens the nanoscale features, reducing the effective surface area and roughness. This morphological evolution provides a clear structural explanation for the wetting behavior. The dense, sharp nanoneedles of the 15-minutes coating are ideal for stabilizing a stable Cassie-Baxter state, leading to the highest WCA and lowest SA. The finer nanowalls (7.5 min) and the coarser nanowalls (22.5 min) are less effective at air entrapment, resulting in higher water adhesion and inferior superhydrophobicity.

**Fig. 6:**
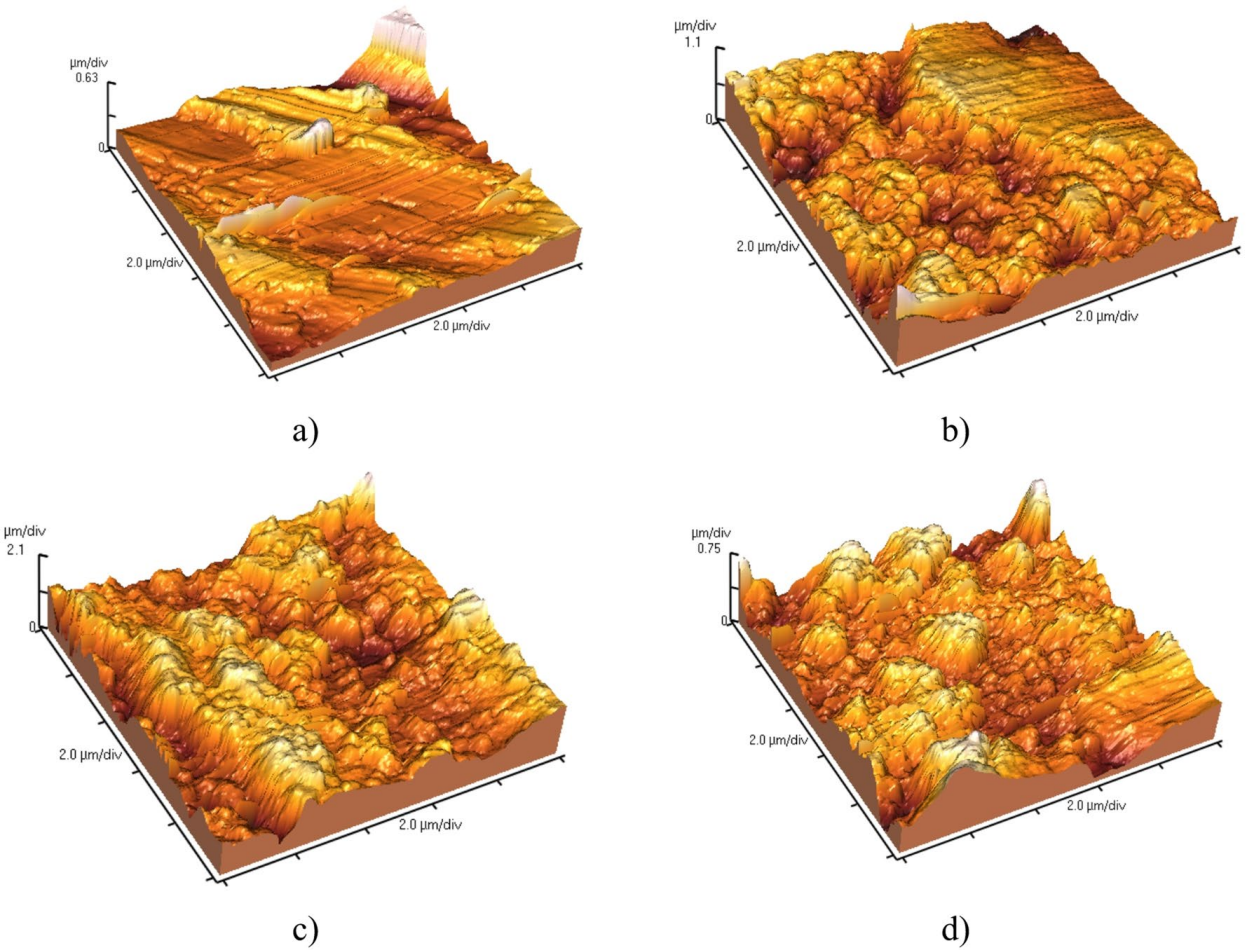
AFM Micrographs of **a**) bare steel and the LDH-SA formed at different deposition times **b**) 7.5 min, **c**) 15 min, and **d**) 22.5 min.

The previous data is confirmed by the AFM measurements, Fig. 6. The polished steel, Fig. 6a, shows the lowest Root Mean Square Roughness (Ra = 1.26 μm), serves as the perfect control. The optimum 15-minutes deposition, Fig. 6c, shows the highest Root Mean Square Roughness (Ra = 4.12 μm). This high Ra value is a direct quantitative measure of the effective surface area and the degree of nanoscale topography created by the LDH. The combination of this high roughness (Ra = 4.12 μm) and the low surface energy from the stearic acid monolayer perfectly satisfies the Cassie-Baxter model. Water droplets sit predominantly on a composite surface of air and the tips of the nanostructures, leading to the observed high WCA and remarkably low SA. The 7.5-minutes sample, Fig. 6b, shows a moderate Root Mean Square Roughness value (Ra = 2.19 μm) which is significantly higher than polished steel but lower than the 15-minutes sample. This confirms that the nanostructured layer is present but not fully developed, resulting in a lower effective roughness. The 22.5-minutes sample, Fig. 6d, shows a moderate Root Mean Square Roughness value (Ra = 1.5 μm). This value is only slightly higher than the polished steel (Ra = 1.26 μm) and indicates low nanoscale roughness and a low superhydrophobicity. The larger size structures, smoother features (low roughness) cannot trap air as effectively. The water droplet makes more intimate contact with solid material, increasing adhesion (higher SA) and reducing the WCA. The surface is transitioning towards a less stable Cassie-Baxter state.

### Corrosion measurements

#### EIS results

**Fig. 7:**
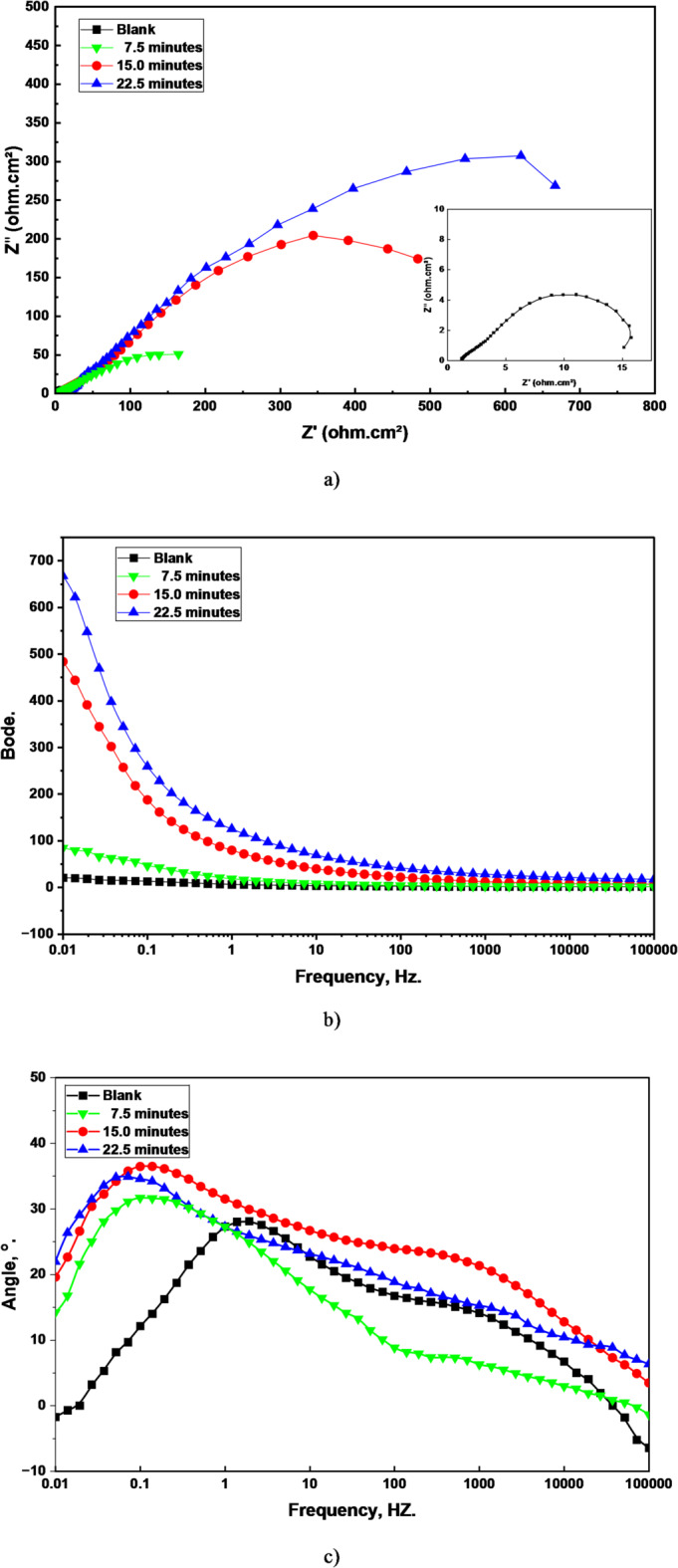
Nyquist (**a**), Bode magnitude (**b**), and phase angle (**c**) plots for bare steel and LDH–SA-coated steel after immersion in 0.5 M NaCl solution. Symbols in Bode magnitude plot represent experimental data, and solid lines represent fitted data using the equivalent circuit.

The EIS data provides a clear and compelling narrative about the corrosion protection characteristics of the prepared LDH-SA coatings, perfectly correlating with the previous structural and wetting analyses. Figure 7 illustrates the Nyquist and Bode plots for bare steel and LDH–SA-coated steel after immersion in 0.5 M NaCl solution. The Nyquist plots (Fig. 7a) exhibit a depressed capacitive semicircle, which is characteristic of the interfacial charge-transfer process at the electrode/electrolyte interface^36^. The LDH-SA coated steel exhibit very high capacitive semicircle. The Nyquist plot’s semicircle diameter is a direct measure of the charge transfer resistance (R_ct_), which stand for the resistance to the electrochemical corrosion reaction at the metal/electrolyte interface. A larger R_ct_ means slower corrosion kinetics.

The bare steel has the smallest R_ct_, indicating a very low inherent resistance to corrosion in the NaCl solution, as expected. The 22.5-minute coating shows a higher R_ct_ than the blank, confirming that even the sub-optimal, overgrown coating provides a physical barrier that slows the access of corrosive Cl^-^ ions and water to the steel surface. However, its R_ct_ is lower than both the 7.5 and 15-minute coatings due to morphological coarsening via Ostwald ripening, which transforms the sharp nanoneedles into larger, smoother nanowalls (as shown in Fig. 5), with no evidence of delamination or cracking, indicating that performance degradation stems purely from morphological coarsening rather than adhesion failure. This reduces surface roughness and compromises superhydrophobicity (WCA decreases from 161° to 156°), thereby diminishing the stability of the air layer that acts as an additional barrier.

The 7.5-minute coating exhibits a higher R_ct_ than the 22.5-minute sample, attributed to its finer nanowall structure and superior superhydrophobicity (WCA = 159°), which enables more effective air entrapment despite incomplete surface coverage. The 15-minute coating (optimum) possesses the highest R_ct_, providing definitive proof of its superior performance. The dense, uniform nanoneedle morphology (average thickness ~ 36 nm) creates an optimally rough hierarchical surface that, combined with the highest superhydrophobic characteristics (WCA = 161°, SA = 3°), establishes a stable Cassie-Baxter state. The trapped air film within the nanostructure acts as an additional insulating layer that drastically hinders the corrosion process, demonstrating that morphological optimization supersedes coating thickness in determining corrosion resistance. So, the corrosion protection performance of the LDH-SA coatings reveals a dual mechanism governed primarily by superhydrophobicity, with coating thickness serving as a secondary physical barrier. The trapped air layer at the solid-liquid interface of superhydrophobic surfaces (Cassie-Baxter state) prevents direct electrolyte contact with the underlying coating and steel substrate, providing the dominant protective effect. This is evidenced by the direct correlation between water contact angle and protection efficiency across all samples. When superhydrophobicity is lost, the coating itself slows corrosive attack through its physical barrier function, though with reduced effectiveness.

The impedance magnitudes at the low frequency in the Bode plots, Fig. 7b, is a direct indicator of the coating’s overall barrier property. The impedance magnitude at low frequency mirrors the R_ct_ trend: Bare steel < 22.5-min < 7.5-min < 15-min. A higher impedance value means better protection. This quantitatively supports the R_ct_ data from the Nyquist plot.

Two distinct time constants are evident at intermediate and low frequencies in the phase angle plot (Fig. 7c). The time constant observed in the low-frequency zone is associated with either the formation of unprotective corrosion products on bare steel or the presence of the protective superhydrophobic coating on the LDH–SA surface. In contrast, the time constant at intermediate frequencies corresponds to the response of the electrical double layer at the metal–electrolyte interface^37–39^. The phase angle equals ~ 35°, this is a classic signature for a coated metal system. A maximum phase angle below 90° indicates a non-ideal capacitive behavior due to the surface heterogeneity and porosity. A value of ~ 35° is consistent with a protective porous layer. A perfect capacitor would show a phase angle of 90°. The fact that it’s not higher suggests the capacitive response is distributed due to the roughness and porosity of the LDH layer. The EIS analysis unequivocally demonstrates that the LDH-SA coatings The optimized coating significantly enhanced the corrosion resistance of steel in 0.5 M NaCl solution.

**Fig. 8:**
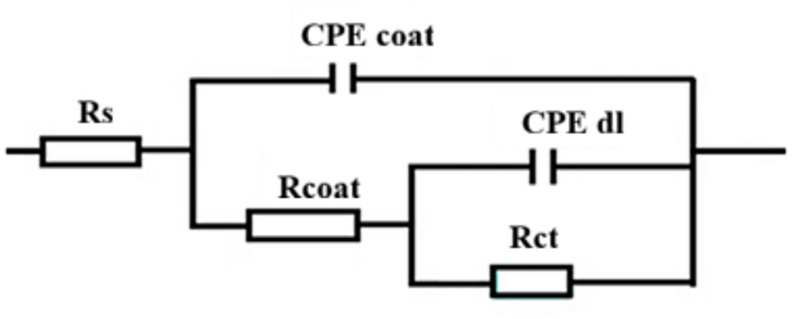
The equivalent circuit model.

The equivalent circuit illustrated in Fig. 8 was employed to fit the experimental data of the EIS, and the impedance parameters were extracted using Zsimpwin software. The quality of fitting was evaluated by Chi-square (χ^2^) values, with small χ^2^ (from 4 × 10^− 5^ to 21 × 10^− 5^) indicating excellent agreement between experimental and fitted data. The circuit includes the following elements: solution resistance (R_s_), coating resistance (R_coat_), coating constant phase element (CPE_coating_), charge-transfer resistance (R_ct_), and the double-layer constant phase element (CPE_dl_). The protection efficiency (%P) was calculated using Eq. (4)^40^:

4 $$\% {\mathrm{P}}={\text{ }}\left[ {\left( {{{\mathrm{R}}_{{\text{ct }}}}{\mathrm{-}}{{\mathrm{R}}_{{\mathrm{ct}}}}^{{\mathrm{o}}}} \right)/{\text{ }}{{\mathrm{R}}_{{\mathrm{ct}}}}} \right]{\text{ X1}}00$$

Rct^o^ and Rct represent the charge transfer resistance for bare steel and the LDH-SA coated steel, respectively. The impedance parameters attained from the equivalent circuit fitting are summarized in Table 1. As evident, both Rct and protection efficiency (%P) follow the trend: Bare steel < 22.5 min < 7.5 min < 15 min, indicating that corrosion resistance improves in the same order. Notably, the LDH-SA coating deposited for 15 min exhibits superior corrosion resistance compared to several previously reported values^41–43^.

**Table 1:** Impedance parameters for bare steel and superhydrophobic LDH–SA-coated steel in a solution of 0.5 M NaCl at 30 °C.

Deposit	*R*_s_ (Ohm.cm^2^)	CPE_coat_x10^5^ (S^*n*^ Ω^−1^ cm^2^)	*n*_1_	*R*_coat_ (Ohm.cm^2^)	CPE_dl_ x10^5^ (S^*n*^ Ω^−1^ cm^2^)	*n*_2_	*R*_ct_ (Ohm.cm^2^)	Χ^2^ (×10^− 5^)	%*P*
Bare steel	1.2 ± 0.10	463.4 ± 5.0	0.64	2.17 ± 0.1	2772.0 ± 10.1	0.67 ± 0.1	13.6 ±0.3	17	--
7.5 min	5.9 ± 0.15	203.3 ± 2.5	0.47	63.9 ± 0.9	311.2 ± 3.2	0.60 ± 0.1	634.2 ± 2.1	4	97.9 ± 0.048
15.0 min	6.1 ± 0.20	178.4 ± 2.0	0.46	76.5 ± 1.6	259.4 ± 2.6	0.66 ± 0.1	911.0 ± 4.1	5	98.5 ± 0.034
22.5 min	4.1 ± 0.12	451.1 ± 1.0	0.50	9.9 ± 0.3	418.1 ± 5.2	0.88 ± 0.1	207.8 ± 1.6	21	93.5 ± 0.153

#### Potentiodynamic polarization results

**Fig. 9:**
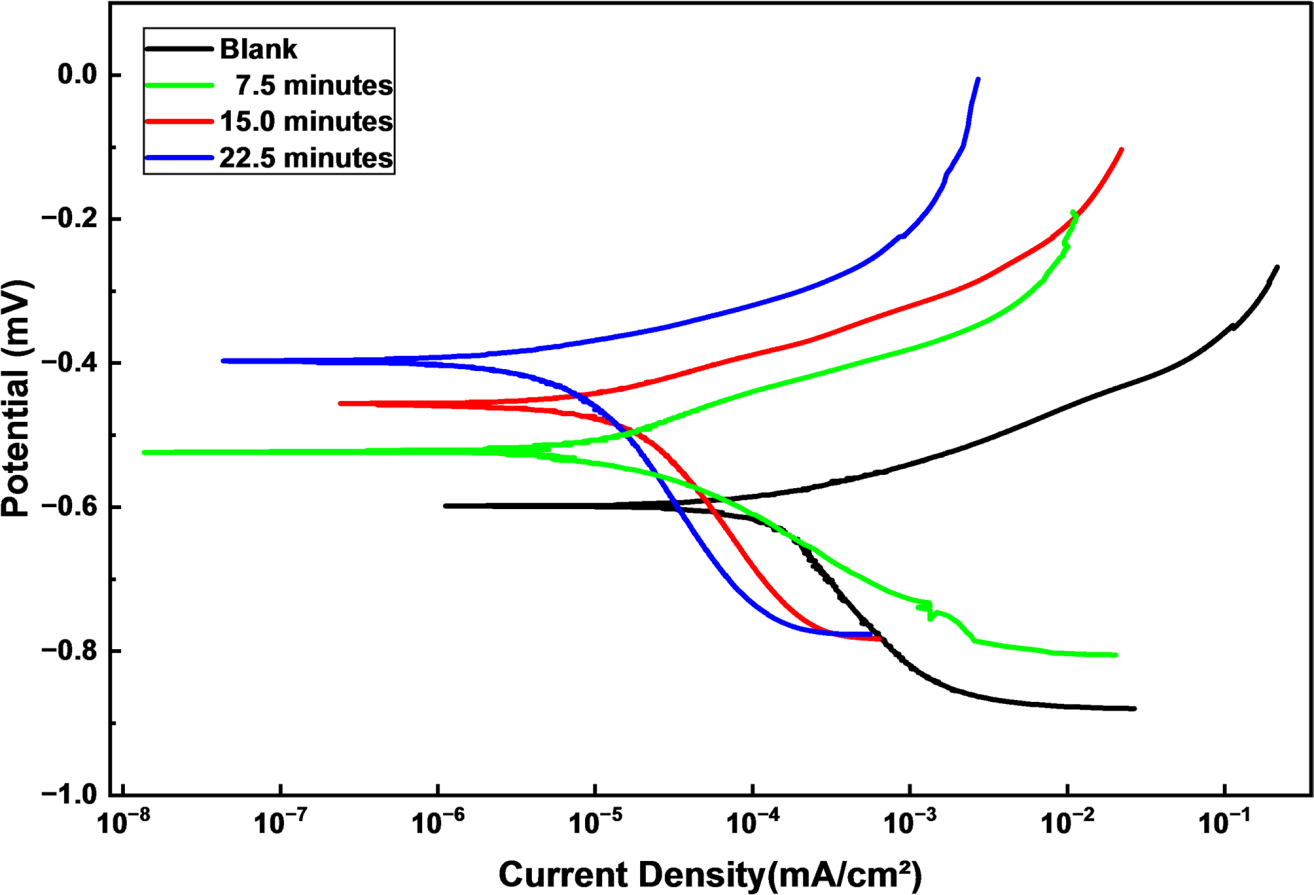
The PDP plots for bare steel and LDH–SA-coated steel in 0.5 M NaCl solution.

The potentiodynamic polarization (PDP) curves, Fig. 9, provide crucial quantitative information about the corrosion kinetics and protective effectiveness of LDH-SA coatings. All three superhydrophobic coatings (7.5, 15.0, and 22.5-minutes deposition) show significant enhancement in corrosion resistance relative to the bare steel substrate, as evidenced by: anodic shift of corrosion potentials (E_corr_) to more noble values and substantial shift of the anodic and cathodic polarization curves to lower corrosion current densities (i_corr_) region. This confirms that coatings effectively hinder both the metal dissolution (anodic) and oxygen reduction (cathodic) reactions that drive corrosion processes.

The potentiodynamic polarization parameters, including corrosion current density (i_corr_), cathodic and anodic Tafel slopes (β_c_ and β_a_), corrosion potential (E_corr_), and protection efficiency (%P) for bare steel and LDH–SA-coated steel, are summarized in Table 2. The protection efficiency (%P) was estimated via Eq. (5)^40^:

**Table 2:** The PDP parameters for bare steel and LDH–SA-coated steel in 0.5 M NaCl solution.

Deposit	-E_corr_ mV	β_a_ mV/decade	-β_c_ mV/decade	i_corr_ µA/cm^2^	%*P*
Bare steel	589.4 ± 2.1	84.8 ± 1.1	232.8 ± 1.6	288.80 ± 1.6	…
7.5 min	457.0 ± 1.8	76.3 ± 0.9	265.5 ± 1.8	19.90 ± 0.4	93.1 ± 0.14
15.0 min	397.6 ± 1.6	69.4 ± 0.7	276.9 ± 2.1	10.05 ± 0.3	96.5 ± 0.11
22.5 min	522.7 ± 1.9	78.6 ± 1.0	103.1 ± 1.2	28.33 ± 0.5	90.2 ± 0.18

5 $$\% {\mathrm{P}}={\text{ }}\left[ {\left( {{{\mathrm{i}}^{\mathrm{o}}}_{{{\mathrm{corr}}.}} - {\text{ }}{{\mathrm{i}}_{{\mathrm{corr}}.}}} \right)/{\text{ }}{{\mathrm{i}}^{\mathrm{o}}}_{{{\mathrm{corr}}.}}} \right]{\text{ }} \times {\text{ 1}}00$$

Where i^o^_corr_. and i_corr_. are the corrosion current densities for bare steel and the as prepared LDH-SA coated steel.

It is obvious that, i_corr_. values for LDH-SA coated steel is markedly lower than that of bare steel. The i_corr_ values follow the trend: 15.0 min < 7.5 min < 22.5 min < bare steel, indicating that corrosion resistance increases in the reverse order.15-minutes coating (optimal): exhibits the lowest corrosion current density (i_corr_) and shows the most positive corrosion potential (E_corr_), demonstrates the strongest suppression of both anodic and cathodic branches. The 7.5-minutes coating shows intermediate protection, while the 22.5-minutes coating shows the lowest protection. The significant reduction in i_corr_ indicates the superhydrophobic layer creates an effective physical barrier against chloride ion penetration. The extremely low current densities, particularly for the 15-minutes coating, suggest the trapped air layer in the hierarchical structure provides additional electrical insulation. Based on typical Tafel analysis of such data, the LDH-SA coating show: Pitting potential shift to more positive values, indicating improved resistance to localized corrosion^44^. This trend is similar to that obtained from the EIS measurements.

### Mechanical stability

**Fig. 10:**
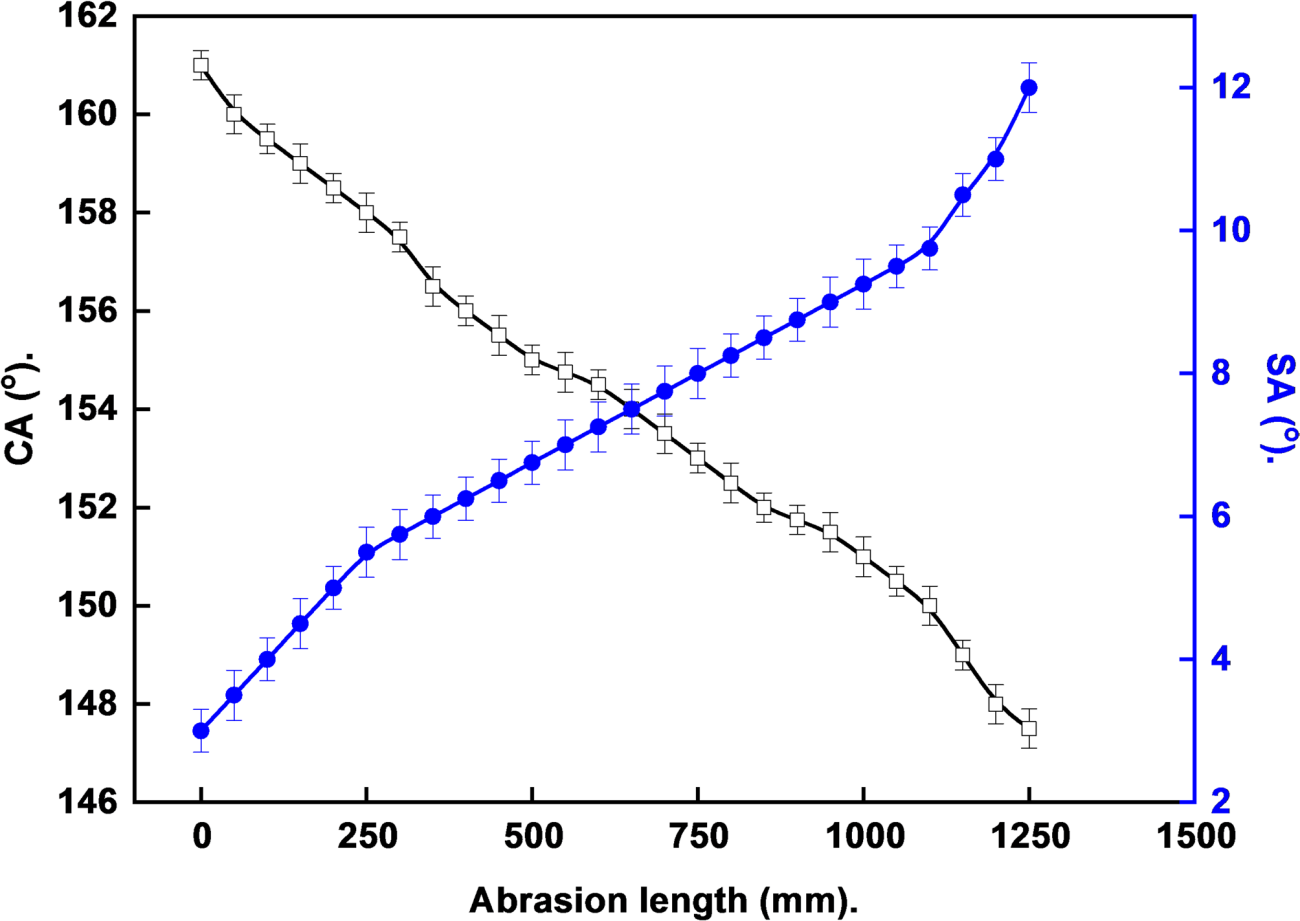
Variation of water contact angle (WCA) and water sliding angle (WSA) as a function of abrasion length for the optimized LDH–SA coating.

The mechanical stability test, Fig. 10, for the optimum LDH-SA coat was performed using abrasion resistance test. The LDH-SA coating retained its superhydrophobic properties up to an abrasion length of 1100 mm, highlighting its remarkable durability and practical applicability. The abrasion test simulates real-world wear and tear. The length (1100 mm) represents the total distance over which a controlled abrasive force (2.5 kPa) was applied before the coating’s superhydrophobicity began to significantly degrade (i.e., WCA fell below 150° and SA rose above 10°). A value of 1100 mm indicates good mechanical resilience. This suggests that the electrodeposited LDH has strong adhesion to the steel substrate and that the nanostructure is not merely a fragile, powder-like layer but a cohesive, integrated coating. The in-situ electrodeposition method ensures chemical bonding between the LDH layer and steel substrate, contributing to its mechanical integrity. The superior mechanical performance stems from the electrodeposition method: in-situ growth creates chemical bonding with the substrate, while the nanoneedle morphology of the optimized 15-minute coating allows crystallite interlocking for mechanical reinforcement. Additionally, the hierarchical structure helps distribute and dissipate applied stress, contributing to the coating’s resilience against abrasive forces. The fact that it withstands such prolonged abrasion while retaining its key property is a major advantage over many superhydrophobic coatings reported in the literature, which often fail under minimal mechanical stress.

**Fig. 11:**
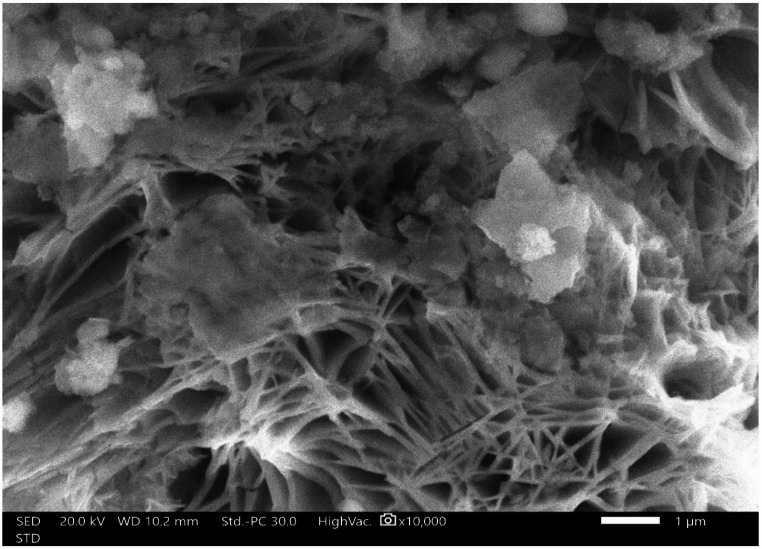
SEM micrograph after the mechanical test for the optimum LDH-SA coat.

The SEM observation, Fig. 11, after the abrasion test is the crucial piece of visual evidence that directly explains the mechanism of the mechanical failure. The SEM micrograph of the coating after the abrasion test provides the definitive structural explanation for the loss of superhydrophobicity after 1100 mm. This observation is not a negative result but a critical and expected part of understanding the coating’s performance limits. The fundamental requirement for achieving superhydrophobicity is the presence of a stable micro/nanostructured surface capable of trapping air pockets. Beyond the 1100 mm point, the mechanical stress is sufficient to physically break down the stearic acid and LDH nanostructure itself, as directly observed in the SEM. The destruction of this nanoscale roughness eliminates the air-trapping capability.

### Chemical stability

**Fig. 12:**
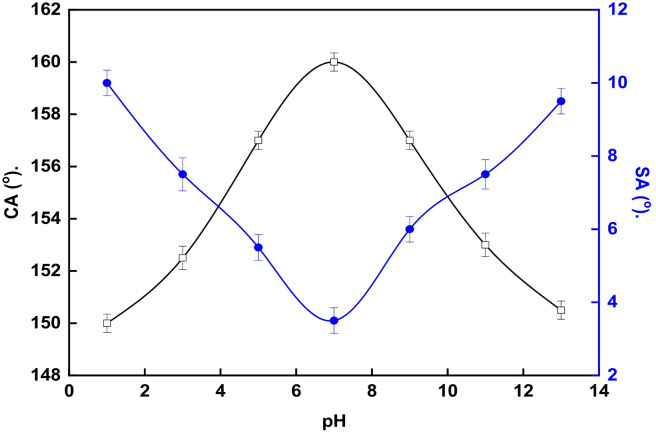
Variation of WCA and SA on LDH–SA coatings following immersion in different pH environments.

The chemical stability for the optimum LDH-SA coat was evaluated for pH range 1–13, Fig. 12. The prepared coat maintains its superhydrophobic properties (WCA > 150°, SA < 10°) across the entire pH range from 1 to 13 which is an exceptional finding. This result demonstrates two key strengths of the coating:

1. Stable chemical inertness: The stearic acid layer grafted onto the LDH is not easily hydrolyzed or desorbed by highly acidic or alkaline solutions. This indicates strong, stable chemical bonding between the stearic acid molecules and the metal cations in the LDH structure, forming a durable, low-surface-energy monolayer that resists harsh chemical environments.
2. Structural integrity of the LDH: The LDH nanostructure itself remains physically intact and does not dissolve or undergo significant structural collapse when exposed to extreme pH. This is crucial because the roughness provided by the LDH is the first pillar of superhydrophobicity. Its stability ensures the air-trapping capability is preserved.

This outstanding chemical stability, combined with the previously demonstrated mechanical abrasion resistance, confirms that this is not just a laboratory curiosity but a technologically viable coating. The fact that it can repel corrosive droplets across the entire pH spectrum suggests its potential for real-world applications.

### Limitations and future perspectives

While the electrodeposition method employed in this study successfully produces superhydrophobic Ni-Al LDH coatings with excellent corrosion protection on steel substrates, certain limitations should be acknowledged regarding its practical applicability. First, the scalability of this process for large-scale or complex-shaped industrial steel structures presents challenges. The uniform distribution of electric field and current density on non-planar or large surfaces may be difficult to achieve, potentially leading to non-uniform coating thickness and morphology. Future work could explore the use of pulse electrodeposition or scanning electrode techniques to improve uniformity on complex geometries, or investigate the feasibility of adapting this method for continuous industrial processing.

Second, the long-term environmental stability of the stearic acid modification layer requires further investigation. Stearic acid, as an organic compound, may undergo photodegradation under UV exposure or gradual deterioration under prolonged outdoor weather conditions. This could potentially compromise superhydrophobicity and corrosion protection over extended periods in real-world applications. Future studies should include accelerated weathering tests (UV exposure, thermal cycling, humidity tests) to evaluate the coating’s durability under realistic outdoor conditions. Additionally, exploring the incorporation of UV-absorbing additives or the development of inorganic low-surface-energy alternatives could enhance the long-term environmental stability of the coating.

## Conclusion

In this study, an environmentally stable superhydrophobic coating was fabricated on a steel substrate via in-situ electrodeposition of Ni–Al layered double hydroxide (LDH), followed by surface modification using eco-friendly stearic acid. The research systematically demonstrated that the electrodeposition time is a critical parameter governing the LDH morphology, which in turn dictates the final wetting and corrosion protection properties. An optimum deposition time of 15 min yielded a uniform layer of nanoneedle LDH structures, producing the highest surface roughness and consequently, the best superhydrophobic performance with a water contact angle of 161° and a sliding angle of 3°.

The superior corrosion protection of this optimum coating was unequivocally confirmed by electrochemical measurements, which showed a significant increase in charge transfer resistance compared to uncoated steel. This was attributed to the superhydrophobicity of the prepared coats, which create a stable composite interface that traps air and acts as an exceptional barrier against corrosive chloride ions. Furthermore, the coating demonstrated outstanding resilience, addressing a major limitation in the field. It withstood extensive mechanical abrasion ( up to 1100 mm) and, most notably, maintained its superhydrophobic state across the entire pH spectrum from 1 to 13. While the coating demonstrated good chemical stability and abrasion resistance, further mechanical stability testing—including tape-peeling and sand-impact tests—remains to be evaluated in future work to fully establish its mechanical durability for practical applications. This environmentally stable chemical stability, combined with its mechanical durability and high corrosion protection efficiency, underscores the practical viability of this coating.

## Data Availability

The data that support the findings of this study are available from the corresponding author upon reasonable request.
